# Liquid–liquid phase separation of the Golgi matrix protein GM130

**DOI:** 10.1002/1873-3468.13715

**Published:** 2019-12-26

**Authors:** Aleksander A. Rebane, Pascal Ziltener, Lauren C. LaMonica, Antonia H. Bauer, Hong Zheng, Iván López‐Montero, Frederic Pincet, James E. Rothman, Andreas M. Ernst

**Affiliations:** ^1^ Department of Cell Biology Yale School of Medicine New Haven CT USA; ^2^ Dto. Química Física Universidad Complutense de Madrid Spain; ^3^ Instituto de Investigación Hospital Doce de Octubre (i+12) Madrid Spain; ^4^ Laboratoire de Physique de l’Ecole Normale Supérieure CNRS PSL Research University Université Paris Diderot Sorbonne Paris Cité Sorbonne Universités UPMC Univ Paris France

**Keywords:** coiled‐coil, Golgi matrix, Golgin, liquid, liquid phase separation

## Abstract

Golgins are an abundant class of peripheral membrane proteins of the Golgi. These very long (50–400 nm) rod‐like proteins initially capture cognate transport vesicles, thus enabling subsequent SNARE‐mediated membrane fusion. Here, we explore the hypothesis that in addition to serving as vesicle tethers, Golgins may also possess the capacity to phase separate and, thereby, contribute to the internal organization of the Golgi. GM130 is the most abundant Golgin at the *cis* Golgi. Remarkably, overexpressed GM130 forms liquid droplets in cells analogous to those described for numerous intrinsically disordered proteins with low complexity sequences, even though GM130 is neither low in complexity nor intrinsically disordered. Virtually pure recombinant GM130 also phase‐separates into dynamic, liquid‐like droplets in close to physiological buffers and at concentrations similar to its estimated local concentration at the *cis* Golgi.

## Graphical abstract

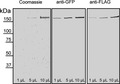

## Abbreviations

**FRAP**, fluorescence recovery after photobleaching

**IDR**, intrinsically disordered regions

The Golgi has long been an object of fascination for cell biologists and still holds many mysteries. Discovered in the late 19th century by Camillo Golgi as a portion of the cell that occasionally took up heavy metal stains, it provided a useful anatomical tool. Over the ensuing decades, the ‘Golgi body’ was found by cytologists in many non‐neuronal cells, especially in glandular cells that produce secretions, and it was gradually recognized to have a likely role in this process, and the term ‘body’ evolved to become ‘apparatus’. With the advent of biological electron microscopy beginning in the 1940s, its ultrastructure proved to be both universal and remarkable: ~ 1‐µm‐diameter stacks of 4–6 flattened, pancake‐like membrane‐bound cisternae surrounded by a swarm of what we now recognize to be 50‐ to 100‐nm‐diameter transport vesicles. The key role of the Golgi apparatus in secretion was firmly established in the 1960s when George Palade and colleagues traced secreted proteins as they traveled from synthesis by ribosomes bound to the ER to the Golgi before reaching secretory storage vesicles 1.

Many secretions contain complex carbohydrates, and most of these sugar residues are added during passage through the Golgi apparatus. The glycosyltransferases catalyzing the successive steps are strategically located in successive cisternae of the stack: Inner sugars in the structure are added mainly at the entry face (termed ‘*cis*’) of the stack; sugars in the middle being added mainly in middle (‘medial’) cisternae; and outermost sugars are mainly added at the opposite (‘*trans*’) end 2. The cargoes then depart from this end after packaging into separate membrane‐bounded carriers, sorted according to their ultimate destination (plasma membrane, lysosome, secretory storage vesicle, etc.).

With the development of GFP‐tagging in the 1990s 3, it became apparent that the Golgi is a highly dynamic structure within which the glycosyltransferases and other membrane proteins—though steady‐state residents—are highly mobile 4. Likewise, the peripheral membrane components of the Golgi constantly exchange with cytoplasmic pools 4. The Golgi extensively fragments prior to cell division, triggered by mitotic phosphorylation, and these fragments of the cisternae re‐assemble in minutes upon de‐phosphorylation within each daughter cell 2, 5. Two classes of drugs acting *via* distinct mechanisms each rapidly disassemble the Golgi, which then spontaneously reappears as rapidly when the drug is washed out 6, 7.

The basis of this remarkable plasticity of this organelle is a long‐standing mystery. How can we account for this dramatic elasticity of this asymmetric structure? An attractive explanation, which we have suggested elsewhere 8, arises from a series of recent discoveries concerning the basis of similar plasticity among so‐called membrane‐less organelles, a category that includes RNA‐containing structures such as P granules, P bodies, cytoplasmic stress granules, and the nucleolus 9, 10, 11, 12, which are now recognized to arise spontaneously by phase separations. Liquid–liquid phase separation of their RNA and intrinsically disordered protein components from cytoplasm (or nucleoplasm) occurs on the basis of numerous low‐affinity mutual interactions that afford each such condensate internal fluidity, a spherical shape, and importantly compositional specificity that is maintained in the steady state in the face of rapid exchange between the condensate and its surrounding medium 13.

If the Golgi were a liquid‐like condensate of cytosol‐derived proteins phase‐separating with membranes (rather than nucleic acids), what could be the identity of these proteins? We have suggested 8 that they may be a class of abundant cytoplasmically derived proteins termed ‘Golgins’ 14 because these proteins are known to bind Golgi membranes and have similar physical–chemical properties, all being helical bundle‐based rods, and because they are located differentially along the *cis‐trans* axis. This latter feature could, in theory, enable internal phase separation to yield Golgi subcompartments, analogous to what has been found for the nucleolus 15.

Golgins are known to function as vesicle ‘tethers’, which initially capture transport vesicles at each level of the Golgi 14. Here, we explore the hypothesis that, in addition to this well‐established function, Golgins may possess the capacity to phase‐separate and in so doing also contribute to the internal, dynamic organization of the Golgi stack. We focused on the most abundant Golgin of the Golgi stack, GM130 16, which is localized at the *cis* face 17. Recent quantitative proteomics by mass spectroscopy indicated that the number of copies of this Golgin in each HeLa cell (about 300 000) exceeds the quantity of its known Golgi anchor (GRASP65) by about 14‐fold 16. We have independently confirmed this by quantitative western blotting (Fig. S1A,B). This raised the interesting possibility that the majority of GM130 could be anchored to the *cis* face of the Golgi indirectly by condensing with the relatively rare copies that are directly bound to the surface.

## Materials and methods

### Cloning of pCMV‐mEGFP‐GM130‐FLAG

The mEGFP‐GM130‐FLAG construct was prepared by PCR amplification of GM130‐FLAG from a Myc‐DDK‐tagged cDNA clone of human GM130 (Origene, RC209641, Rockville, MD, USA) using the forward primer CTCAAGCTTCGAATTCTGGTAGTCTGGAAGTTCTGTTCCAGGGGCCGC

TGATGTGGCCCCAACCCCGCCTCC and the reverse primer GTCGACTGCAGAATTAAAC

CTTATCGTCGTCATCCTTGTAATCCAGGATATCA. The amplification product was purified using the QIAquick gel‐extraction kit (Qiagen, 28704, Hilden, Germany) and subcloned into an EcoRI‐digested (NEB, R3101S) mEGFP mammalian expression vector (Addgene plasmid #54759, gift from Michael Davidson) using In‐Fusion Cloning (Takara Bio, 638910, Kusatsu, Japan).

### Cell culture, transfection, and labeling

HeLa cells (ATCC, CCL‐2, Old Town Manassas, VA, USA) were grown at 37° C and 5% CO_2_ in Dulbecco’s modified Eagle’s medium (Thermo Fisher Scientific, Waltham, MA, USA) supplemented with 10% FBS (Thermo Fisher Scientific). 10^6^ cells were electroporated with 4 µg GFP‐GM130 using Nucleofector Kit R (Lonza, VVCA‐1001, Basel, Switzerland) and program I‐13 on a Nucleofector 2b device (Lonza, AAB‐1001). Cells were seeded on a glass‐bottom dish (MatTek, P35G‐1.5‐14‐C, Ashland, MA, USA) coated with fibronectin (Millipore, FC010, Burlington, MA, USA) for live‐cell imaging, or on fibronectin‐coated coverglass (Electron Microscopy Sciences #1.5, 12 mm), and fixed by incubating 15 min with 4% para‐formaldehyde. Fixed cells were washed with PBS and permeabilized with permeabilization buffer (0.05% Triton X‐100, 0.3% IGEPAL CA‐630, 0.1% BSA in PBS) for 3 min, then washed with wash buffer (0.05% Triton X‐100, 0.05% Igepal CA‐630, 0.2% BSA in PBS), and blocked 1 h in blocking buffer (0.05% Triton X‐100, 0.05% Igepal CA‐630, 5% normal goat serum) at room temperature. Cells were labeled with anti‐GM130 (BD, 610823, Franklin Lakes, NJ, USA) for 1 h, washed and labeled with Alexa Fluor 647 secondary antibody (Thermo Fisher Scientific, A‐21236), and washed and then mounted in prolong gold (Invitrogen, P36930, Carlsbad, CA, USA). Cells were imaged on a Zeiss (Oberkochen, Germany) LSM 880 Airyscan confocal microscope.

### Fluorescence recovery after photobleaching (FRAP) protocol and analysis

HeLa cells were grown and transfected with mEGFP‐GM130‐FLAG as described above. FRAP experiments were performed on a Zeiss LSM 880 Airyscan confocal microscope, using a Plan‐Apochromat 63× Oil objective (numerical aperture = 1.4) and an acquisition rate of 2.5 frames per second. Fluorescence photobleaching and recovery were conducted using λ_ex_ = 488 nm and λ_em_ = 500–580 nm, with one scan at 100% laser power for bleaching, and by monitoring recovery at 2% of the maximum excitation laser power. Recovery curves were fitted as previously described 18, using Wolfram Mathematica, and by setting the last bleaching frame as *t* = 0 of the fluorescence recovery curve.

### Protein purification from Expi293 cells

The mEGFP‐GM130‐FLAG plasmid was transfected into Expi293 cells (Thermo Fisher) at 1 µg·mL^−1^ culture, employing poly(ethylenimine) (µL) : DNA (µg) ratio of 3 : 1; 6 h post‐transfection, growth enhancers were added according to the instructions of the manufacturer. After 48 h, the cells were pelleted at 500 ***g*** for 15 min and washed with PBS. Typically, a pellet stemming from a 150 mL culture was resuspended in 15 mL buffer 1 [50 mm HEPES/KOH pH 7.3, 175 mm NaCl, 5 mm EDTA, 1 mm PMSF, 1 mm TCEP, protease inhibitor tablet (Roche, Basel, Switzerland)]. Next, 0.33% Triton X‐100 (v/v) was added and the lysate rotated at room temperature for 20 min. After adding buffer 1–50 mL and another 20 min of incubation, unlysed material was pelleted at 16 500 ***g*** for 15 min at 12 °C. Next, 4.5‐mL anti‐FLAG affinity resin was washed with 20 mL buffer 1, followed by a wash with 10 mL buffer 1 containing 0.1% (v/v) Triton X‐100. Next, the lysate was added to the washed beads and incubated for 3 h at room temperature. The suspension was settled on a column and drained, and washed with 10 mL buffer 1. Next, the beads were washed with 50 mL buffer 2 (buffer 1 plus 1 mm ATP, 1 mm MgCl_2_). Recombinant GM130 was eluted from the beads in buffer 3 (buffer 1 plus 230 ng·mL^−1^ FLAG peptide), 35 min per elution, six fractions total. Immediately after elution, the fractions were spun at 10 000 ***g*** for 6 min and the supernatant desalted on G25‐Sephadex (Thermo Fisher, NAP‐5) columns equilibrated with 5 mm HEPES/KOH pH 7.3. The concentration of recombinant mGFP‐GM130‐FLAG was determined by quantitative western blotting employing a recombinant GFP standard with known concentration (Abcam, Cambridge, UK). To determine the concentration of GM130 in cells, recombinant GFP‐GM130‐FLAG was blotted at increasing (known) protein amounts and compared to lysates of increasing amounts of EXPI293F cells that were subjected to automated cell counting (Bio‐Rad, TC20, Hercules, CA, USA). Statistical analysis was performed using graphpad prism 6 (GraphPad Software, San Diego, CA, USA) for unpaired, two‐tailed *t*‐tests. Differences were considered significant if *P*‐value < 0.05(∗), <0.01(∗∗), or < 0.001(∗∗∗).

### Concentration measurements of phase‐separated GM130 using fluorescence intensity

The sample of purified mEGFP‐GM130‐FLAG was pipetted onto a glass‐bottom dish (MatTek, P35G‐1.5‐14‐C) and imaged using a Zeiss LSM 710 Duo confocal microscope, with the experimental conditions held constant across measurements. The acquired images were quantified using imagej (NIH, Bethesda, MD, USA) by calculating the mean and standard deviation of pixel intensity within a region of interest 19. ROIs for individual GM130 condensates were obtained using the Analyze Particles Tool in imagej, yielding distinct intensity and size measurements for several hundred condensates. Importantly, the mean pixel intensity was found to be independent of condensate size for diameters > 500 nm, consistent with their large size relative to the confocal volume. Smaller condensates were not included in the subsequent analysis because their intensity is expected to depend on their location relative to the confocal volume. The calibration curve relating the pixel intensity to fluorophore concentration was obtained by imaging recombinant 6xHis‐mEGFP‐FLAG solutions of different known concentrations. Fluorescence intensity in the calibration images was found to depend on the distance of the imaging plane from the glass surface. In order to account for this systematic error, calibration images were acquired at ten evenly spaced heights ranging 0–30 µm from the glass surface, and their mean pixel intensities averaged. The resultant intensities were plotted as a function of mEGFP concentration and fitted with a linear function using Wolfram Mathematica, taking into account the uncertainties stemming from variations between individual measurements and the systematic error arising from the height dependence. Using this calibration curve, the mean pixel intensities of GM130 condensates were converted to concentrations. The measurement uncertainties were calculated from intensity variations between individual condensates and the previously determined uncertainty of the best‐fit calibration curve. Similarly, the critical concentration for GM130 phase separation was obtained by drawing ROIs around phase transition events and measuring their pixel intensities immediately prior to the transition event.

## Results and Discussion

### Overexpressed GM130 forms liquid‐like droplets in cells

One hallmark of an intrinsically disordered protein that forms or contributes to membrane‐less organelles is its individual capacity to phase‐separate into micron‐scale condensates when overexpressed in cells. Typically, these condensates are spherical at first and are referred to as ‘droplets’ because of their dynamic behavior. Their constituents diffuse within a droplet; they exchange among droplets, and the droplets themselves coalesce by fusion over time. Although the droplets behave as liquids at the outset, over minutes to hours they typically ‘harden’ as the condensed protein molecules form increasingly static, solid‐like arrangements. This becomes evident as the condensates gradually assume nonplastic, irregular shapes and the other liquid‐like properties diminish 20.

**Figure 1:**
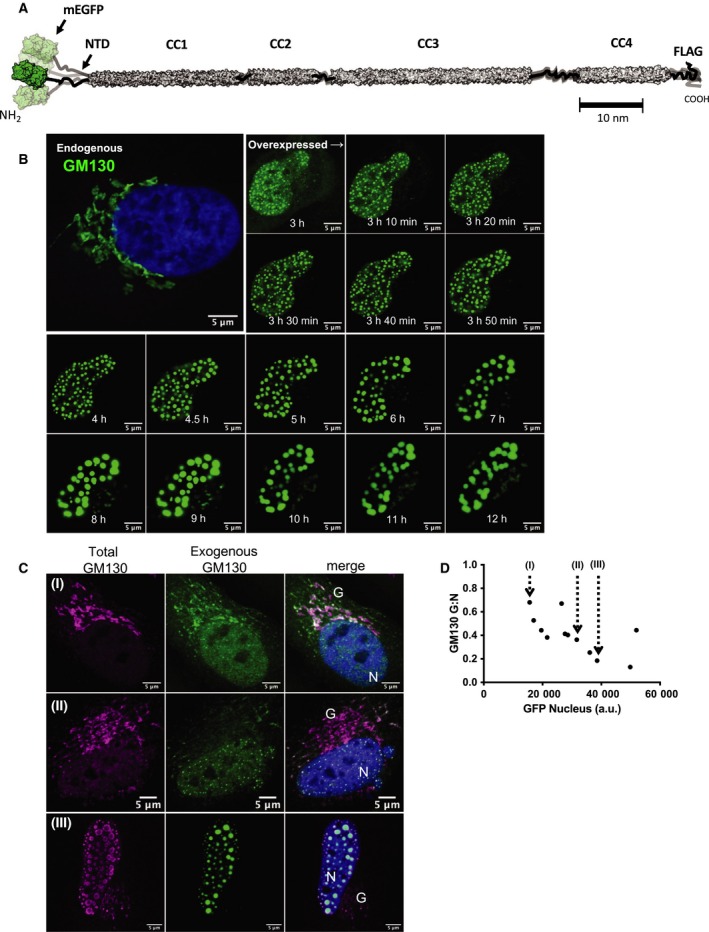
GM130 forms condensates in the nucleus. (A) Tetrameric structure of recombinant mEGFP‐GM130‐FLAG obtained by computational modeling. The C‐terminal FLAG tag was used for affinity purification in subsequent experiments (see main text). (B) Upper left: localization of endogenous GM130 (immunofluorescence: green; nucleus: blue). Gallery: time‐lapse confocal imaging of a single representative HeLa cell transfected with mEGFP‐GM130‐FLAG. (C) mEGFP‐GM130‐FLAG‐transfected HeLa cells were fixed 3–6 h post‐transfection and imaged for total GM130 (anti‐GM130 immunofluorescence) and exogenous GM130 (GFP fluorescence). i), ii), and iii) represent cells with low, medium, and high mEGFP‐GM130‐FLAG expression levels, respectively (max. intensity projections of Z‐stacks). (D) The ratio of total GM130 in the Golgi (G) versus the nucleus (N) was plotted as a function of GFP signal in the nucleus. Scale bars: 5 µm.

The Golgin GM130 is a homo‐tetramer of ~ 130 kD subunits consisting of four parallel coiled‐coil segments with interspersed flexible linkers, and with short nonhelical regions at both its amino and carboxyl‐terminal ends (Fig. 1A). When fully extended, GM130 can potentially extend to ~ 100 nm 21. A GFP‐tagged version of GM130 (also containing a FLAG epitope tag for subsequent purification) was overexpressed in HeLa cells and studied by confocal microscopy (Fig. 1B). Surprisingly, because GM130 lacks abundant low complexity sequences and is not intrinsically disordered, condensates form, and these occur mainly in the nucleus. GM130 contains a nuclear localization signal near its amino‐terminus 22. Ordinarily, this signal is blocked by binding to another Golgi‐related protein p115 22, but when GM130 is overexpressed and exceeds its partner, the excess unbound GM130 is expected to be taken up by the nucleus.

The abundance, size, and properties of the GM130 condensates evolved over time. At 3 h post‐transfection, GM130 had already accumulated in the nucleus, forming a ‘haze’ of uncondensed protein interspersed with condensed, punctate structures (Fig. 1B). Over the course of the next hour, the background haze gradually diminished as the size of the condensates grew. By 4 h, the vast majority of nuclear GM130 had assembled into apparently spherical, droplet‐like structures ~ 1 µm in diameter. These spherical condensates continued to grow in size up to ~ 2 µm in diameter over the next several hours and increasingly became nonspherical, suggestive of hardening.

The simplest interpretation is that free GM130 tetramers are transported across nuclear pores into the nucleoplasm, forming the background haze. Over time, the concentration of GM130 in the nucleus progressively increases, and when it saturates, the separate droplet phase begins to form. Additional GM130 then accrues to the droplets, which grow correspondingly in size. The droplets not only grow in size, but also become reduced in number (compare, e.g., 8 h with 4 h in Fig. 1B) as they coalesce by fusion, which will be studied in detail below.

How relevant is the formation of the nuclear condensates of overexpressed GM130 to the physiological mechanism of association of endogenous GM130 in the Golgi? The fact that the condensates of overexpressed GM130 accumulate inside the nucleus, while the Golgi itself remains outside the nucleus creates a fortuitous opportunity to answer this question, using a form of the classic competitive binding experiment in which the distribution of a common ligand across a dialysis membrane between two partners measures their relative affinity for the ligand 23. Here, the nuclear envelope plays the role of the dialysis bag, and the GM130 tetramer is the ligand, partitioning across the membrane between Golgi and condensates. If the endogenous Golgi complement of GM130 re‐distributes to the nuclear condensates, then the energetic environment associating them to condensates must be similar or even more favorable than that retaining it in the Golgi.

To test this, we transfected HeLa cells with GFP‐GM130 for 3–6 h before the cells were fixed and stained with anti‐GM130 antibodies to equally reveal the total of both expressed exogenous and endogenous GM130 (anti‐GM130), or by imaging GFP fluorescence to selectively reveal the exogenous population (Fig. 1C). In cells with low exogenous GFP‐GM130 expression levels i), the majority of the total GM130 remained localized to the Golgi, while the exogenous GM130 localized to Golgi but also accumulated in the nucleus, mainly as the background ‘haze’ of apparently unassembled tetramers. At intermediate expression levels ii), the exogenous GM130 population is mainly in the nucleus and droplets containing exogenous GM130 begin to appear in the nucleus. At high expression levels iii), the majority of the total (exogenous plus endogenous) GM130 mainly resides in spherical droplets within the nucleus; little GM130 remains at the Golgi. The proportion of total GM130 in the Golgi decreases continuously as a function of increasing GFP‐GM130 expression levels (Fig. 1D). This depletion of GM130 from the Golgi area by relocation results in Golgi fragmentation (as judged from immunolabeling of the cis Golgi resident and membrane‐integral protein GPP130 24; Fig. S1C), corresponding to the phenotype when endogenous GM130 is knocked down with siRNA 25. Note that the spherical GM130 nuclear condensates revealed by GFP typically have only their outer surfaces stained by anti‐GM130 antibodies, giving them a ring‐like appearance (Fig. 1C, iii). This suggests that the antibodies could not fully penetrate the condensates after fixation and permeabilization of the cells.

Our results so far indicate that excess GM130 is transported into the nucleus to form spherical condensates that are apparently liquid‐like on the basis of their shape. These droplets must create an energetically similar microenvironment to that experienced by endogenous GM130 as it resides in the *cis* Golgi, because the endogenous Golgin can favorably join these droplets when they are present in excess.

**Figure 2:**
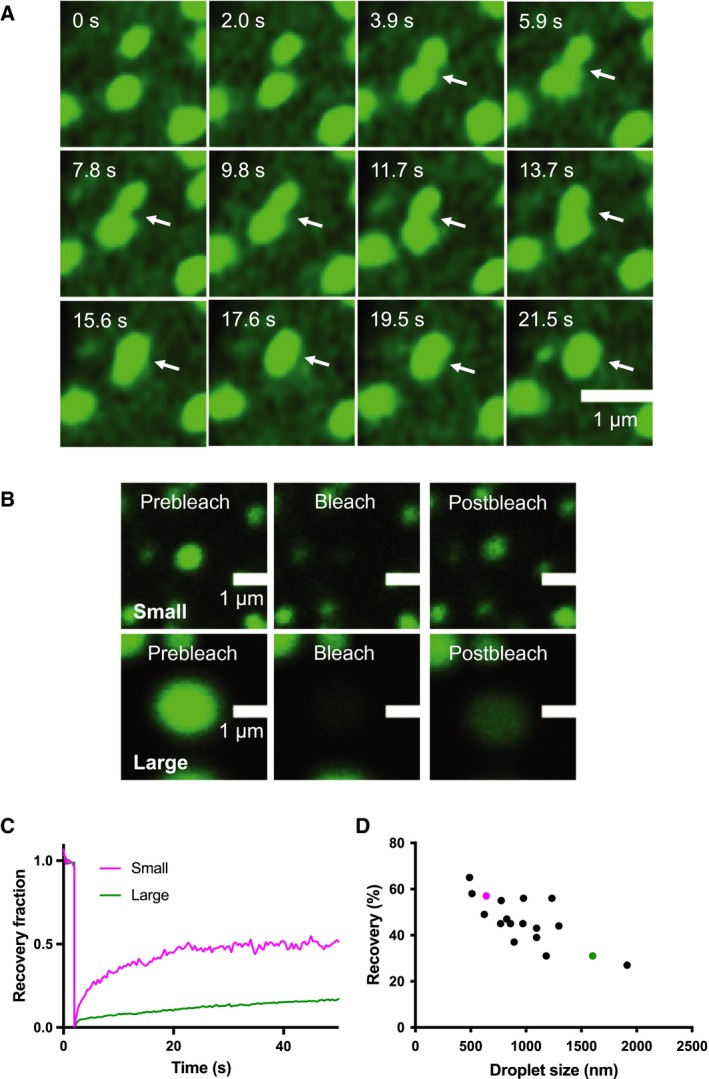
Phase‐separated GM130 condensates in the nucleus are dynamic. (A) Live‐cell confocal microscopy of a fusion event (white arrows) between two nuclear mEGFP‐GM130‐FLAG condensates. (B) Representative FRAP of small (upper panel) and large (lower panel) mEGFP‐GM130‐FLAG nuclear condensates imaged 2–5 h post‐transfection. (C) Representative rate of recovery of small (magenta) and large (green) nuclear mEGFP‐GM130‐FLAG condensates. (D) Mobile fraction (% recovery) of nuclear mEGFP‐GM130‐FLAG droplets is plotted as a function of their size. Highlighted data points correspond to images shown in (C). Scale bars: 1 µm.

To directly assess fluidity, we looked for fusion among droplets and internal diffusion of their constituents, the current standard in the field 26. At 3 h post‐transfection, when the droplets are prominent and still primarily spherical, we observed fusion events in which spherical droplets coalesced and relaxed back into a combined spherical shape within a few seconds (Fig. 2A). We investigated the mobility of GM130 within the droplets by performing fluorescence recovery after photobleaching (FRAP) experiments on individual nuclear condensates (Fig 2B,C). Smaller and presumably younger condensates (< 1 µm diameter) exhibited a faster rate of recovery and a greater mobile fraction than the larger and presumably older condensates (> 1 µm diameter). In fact, we observed an almost linear relationship between the size of GM130 nuclear droplets and their mobile fractions, with the mobile fraction dropping below 50% for droplets exceeding 1 µm in diameter (Fig. 2D).

### Purified GM130 phase‐separates into dynamic, liquid‐like droplets

**Figure 3:**
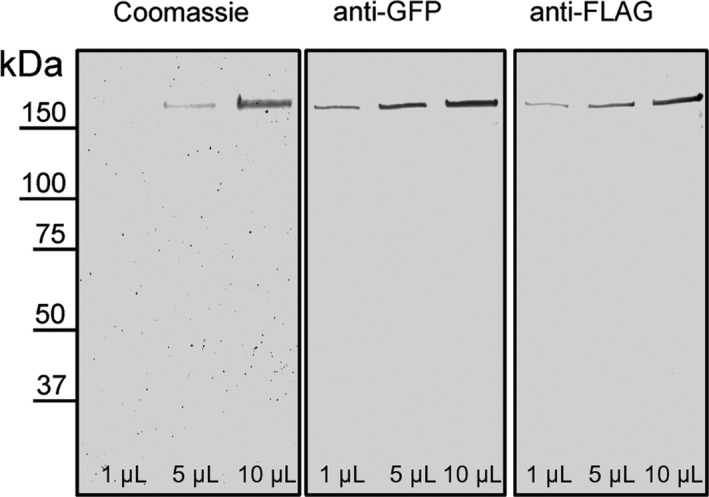
Purity of recombinant mEGFP‐GM130‐FLAG. SDS/PAGE of purified mEGFP‐GM130‐FLAG stained with Coomassie (left), and the accompanying western blots using anti‐GFP (middle) and anti‐FLAG (right) antibodies.

**Figure 4:**
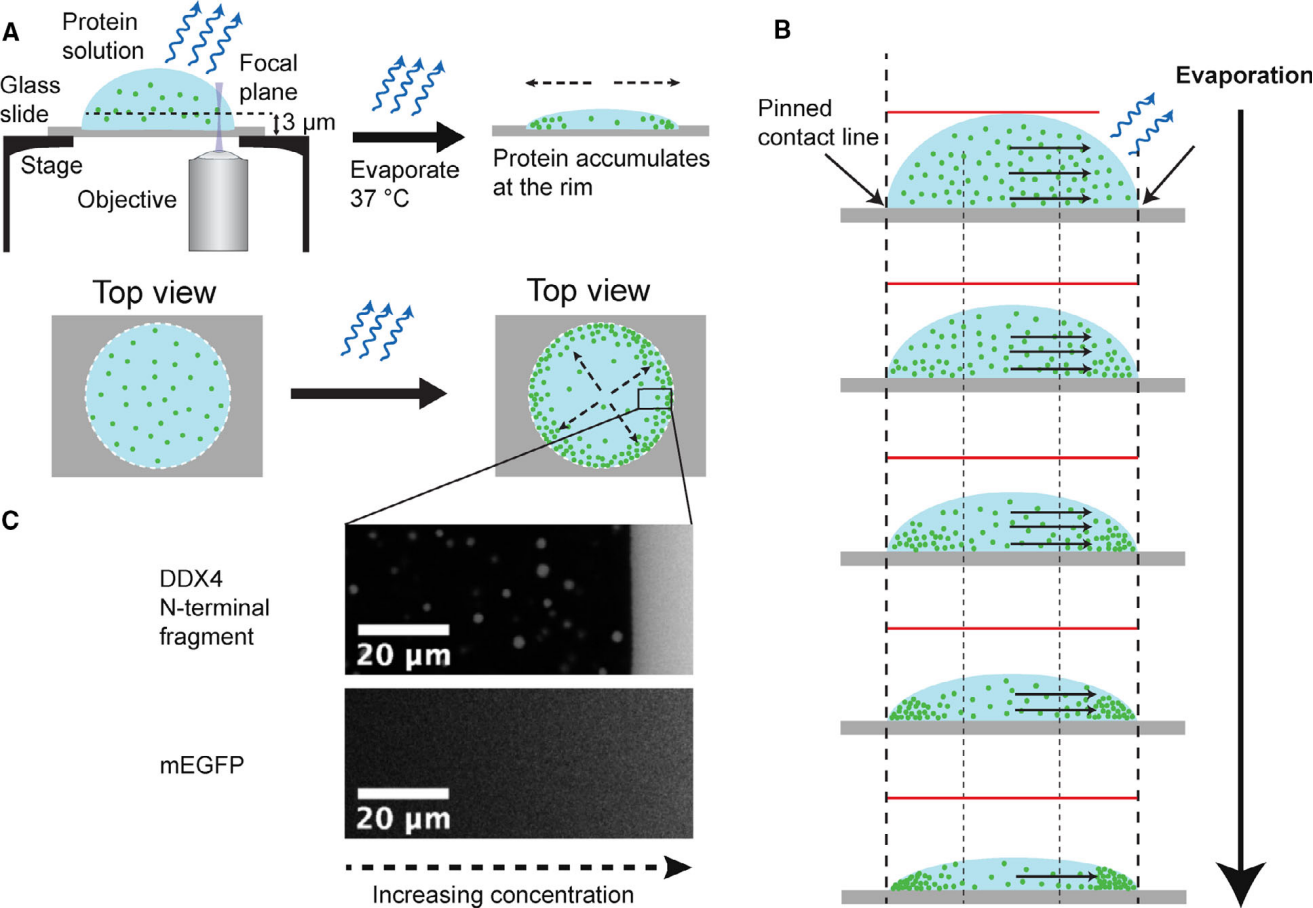
Illustration and validation of the evaporation assay. (A) Illustration depicting confocal fluorescence imaging of a drop of mEGFP‐GM130‐FLAG solution during evaporation. The protein accumulates at the rim of the drop due to the coffee ring effect, forming a concentration gradient that increases over time. In all experiments, a focal plane 3 µm above the glass slide was chosen to minimize fluorescence background. (B) Illustration of the coffee ring effect in an evaporating drop of protein solution with pinned contact line to the glass slide. Evaporated fluid near the contact line must be replenished and by an outward flow of solution from the interior, thereby concentrating the protein near the rim of the drop. (C) Time‐lapse of accumulation of protein at the edge of the protein solution during evaporation. Upon evaporation, Alexa Fluor 647‐labeled recombinant N‐terminal domain of Ddx4 (residues 1–236) (5 mm HEPES/KOH pH 7.3, 120 mm KCl, and 1 mm TCEP and at 37 °C) forms spherical condensates (top panel), whereas recombinant 6xHis‐mEGFP‐FLAG (20 mm HEPES/KOH pH 7.3, 140 mm KCl, 1 mm MgCl_2_, 1 mm DTT, and at 37 °C) forms a smooth concentration gradient (bottom panel). The dashed arrow points along the gradient of increasing protein concentration, from the center of the evaporating sample toward its rim. Scale bars: 20 µm.

**Figure 5:**
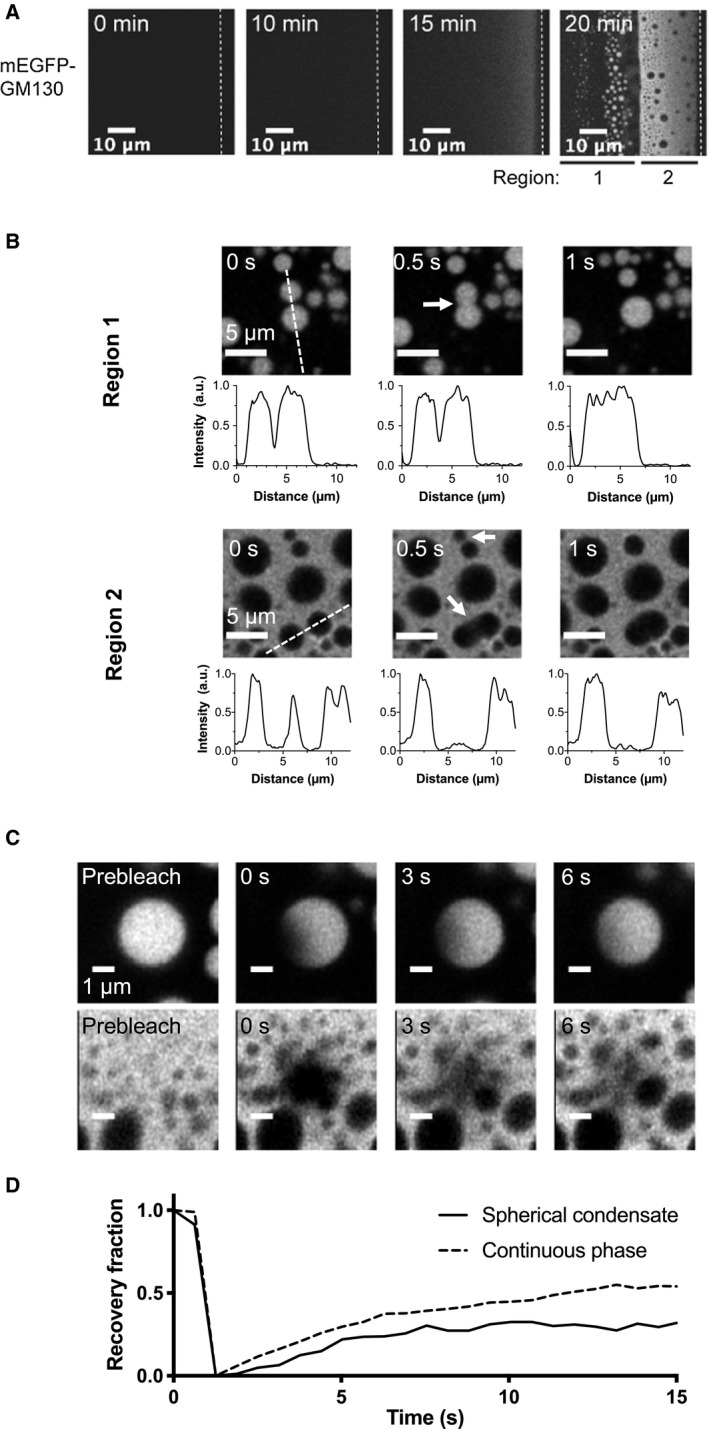
Purified GM130 phase‐separates into liquid‐like condensates *in vitro*. (A) Time‐lapse of accumulation of mEGFP fluorescence at the edge of the protein solution during evaporation (20 mm HEPES/KOH pH 7.3, 140 mm KCl, 1 mm MgCl_2_, 1 mm DTT, and at 37 °C). Upon evaporation, recombinant mEGFP‐GM130‐FLAG forms a gradient and then becomes supersaturated at around 20 min, undergoing liquid–liquid phase separation. At intermediate concentration (Region 1), mEGFP‐GM130‐FLAG phase‐separates into spherical condensates whereas at high concentration (Region 2), it forms a continuous dense phase containing fenestrations of dilute phase where protein is depleted. (B) Time series showing fusion between two condensates (Region 1) or two fenestrations (Region 2). White arrows indicate individual fusion events. Line scans indicate fluorescence intensity along the dotted white line for each frame. Scale bars: 5 µm. (C) Representative FRAP of a spherical condensate (upper panel) and the continuous phase (lower panel) of phase‐separated mEGFP‐GM130‐FLAG. Scale bars: 1 µm. (D) FRAP curves of the spherical condensate (solid) and the continuous phase (dashed) shown in (C).

To test for possible phase separation of GM130 *in vitro*, we established a protocol enabling the reliable and reproducible purification of native mEGFP‐tagged GM130 expressed in Expi293F cells whose purity exceeded 95% as determined by quantitative SDS/PAGE and western blotting (Fig. 3). We then validated an assay 27 (Fig. 4A) to test for phase separation of these recombinant proteins. A 5–10 µL drop of buffered protein solution was deposited on an exposed microscope slide and allowed to evaporate, thereby inducing advective flows that gradually concentrated the protein near the rim of the drop in what is termed the coffee ring effect 28 (Fig. 4B). These flows arise because the contact line, at which the drop surface meets the microscope slide, is held in place or ‘pinned’ by surface roughness and chemical heterogeneities in the glass substrate. Consequently, whereas the evaporative loss near the drop center will merely decrease the drop height, evaporated fluid near the contact line must be replenished by an outward flow of fluid (Video S1). We monitored this process using confocal microscopy of mEGFP fluorescence and found that over the course of 10–20 min of evaporation, the local protein concentration at the rim increased (Fig. 4C), in agreement with numerical modeling (Appendix S1). As negative controls, neither recombinant purified mEGFP alone (nor a number of other control proteins) condensed under the same conditions (Fig. 4C lower panel and Fig. S2), whereas a well‐characterized phase‐separating protein, the disordered N terminus of Ddx4 29, did condense (Fig. 4C, upper panel). These results establish the ability of our assay to faithfully probe phase separations. Samples containing 10–100 nm GM130 were then tested at 37 °C. As the local concentration of GM130 increases near the rim of the sample, the protein spontaneously condenses (Fig. 5A) into a multitude of µm‐sized droplets (region 1) and at even higher concentrations into an inverted phase in which the GM130‐rich phase is continuous and hosts aqueous droplets depleted in GM130 (region 2). This morphology is in agreement with theoretical predictions for phase separation in concentration gradients 30. Importantly, the droplets appear within 1 s and all at once, indicative of crossing a phase transition boundary (compare Fig. 5A, 15 and 20 min, and Video S2 at time 914 s). The droplet assay further revealed that purified mCherry‐tagged fragments of GM130, several of which were predicted to form coiled‐coils in their entirety 31, could also undergo phase separation, albeit to different extents (Fig. S3). Importantly, concerning the above experiments we generally employed conditions approximating the cytoplasm (20 mm HEPES/KOH, pH 7.3, 140 mm KCl, 1 mm MgCl_2_, 1 mm DTT) and notably did not add any crowding agent (such as a polyethylene glycol) needed in many other cases to observe condensate formation *in vitro* 26, 32, 33. The droplet evaporation assay yielded similar results even with a simplified buffer composition (5 mm HEPES/KOH, pH 7.3, 140 mm KCl), thereby ruling out that phase separation is induced by increased concentrations of buffer components at the rim. In subsequent experiments, we therefore employed the simplified system to minimize the concentration gradients arising from the various buffer components during evaporation. To be certain that the condensates were not due to excessive local concentrations of salts in regions 1 and 2, we represent the droplet evaporation assay with initial [KCl] in the range from 0 to 500 mm with qualitatively similar results across the entire range (Fig. S4).

As observed for the spherical droplets of GM130 in the nucleus, both the droplets rich in GM130 in region 1 and the aqueous droplets hosted by the continuous GM130 phase in region 2 coalesced *via* fusion and relaxed back to into spherical shapes thereafter (Fig. 5B). We investigated the mobility of GM130 in individual droplets and the continuous phase using FRAP experiments (Fig. 5C,D). The individual droplets and the continuous phase of GM130 both exhibited fluorescence recovery within seconds of photobleaching.

**Figure 6:**
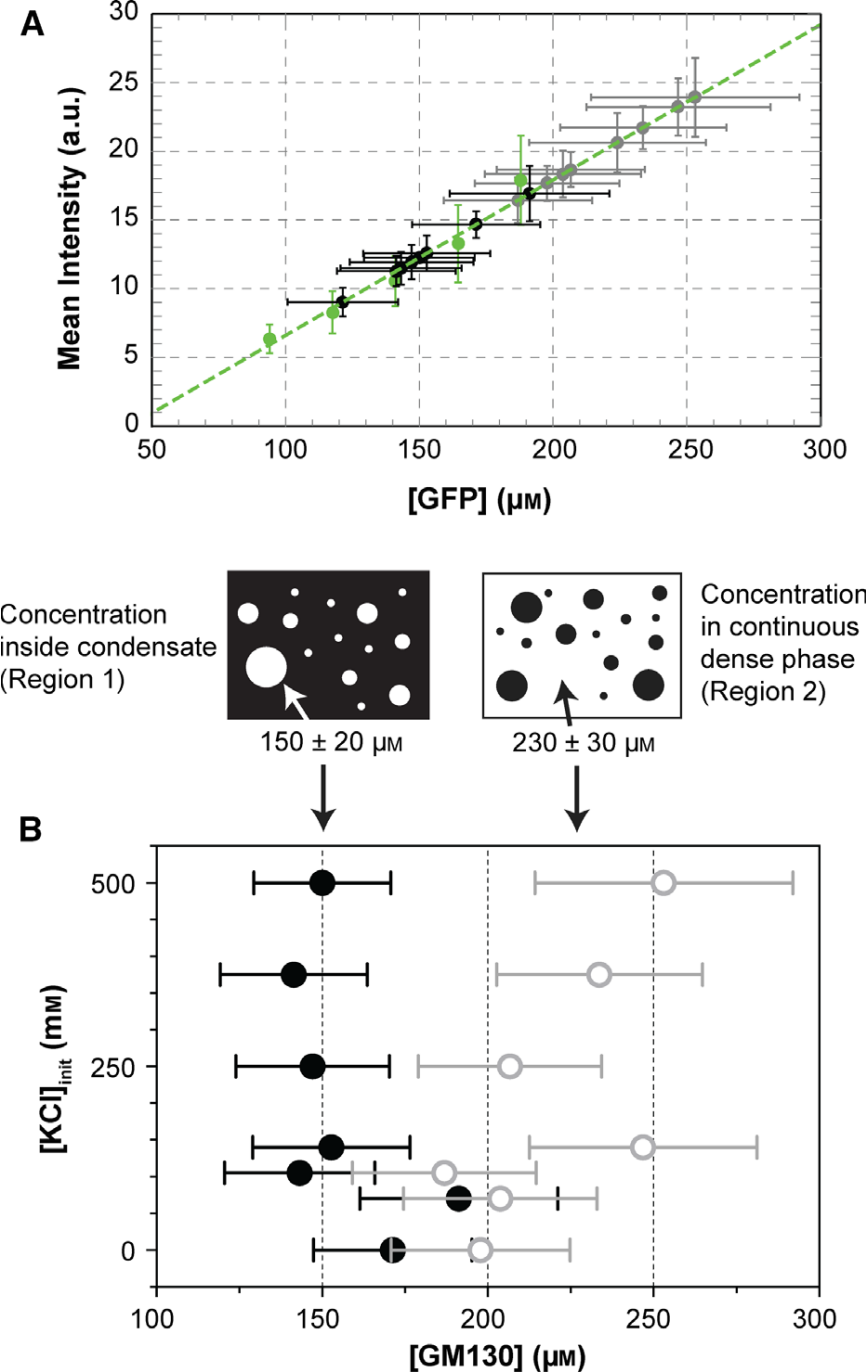
Concentration measurements of phase‐separated GM130 *in vitro*. (A) mEGFP‐GM130‐FLAG concentration measurements shown in (B) fall into a well‐calibrated range for the mEGFP standard curve. Green points denote measurements of mEGFP fluorescence intensity for solutions of known mEGFP concentrations at 37 °C in 5 mm HEPES/KOH pH 7.3, 140 mm KCl. Error bars indicate systematic error in fluorescence intensity measurement due to variations in microscope optics. The green line is the fitted calibration curve. Black and gray points denote fluorescence intensity measurements of mEGFP‐GM130‐FLAG condensates and the continuous dense phase, respectively. Vertical error bars stem from variability between individual fluorescence intensity measurements. Horizontal error bars represent the standard error in mEGFP‐GM130‐FLAG concentration calculated from the uncertainty in the calibration curve and the variability between individual measurements. (B) Concentration measurements of mEGFP‐GM130‐FLAG inside condensates (solid black circles) and in the continuous dense phase (open gray circles) for buffers containing 5 mm HEPES and different initial salt concentrations at the onset of evaporation and at 37 °C. The concentration values were calculated from mEGFP fluorescence using the mEGFP calibration curve shown in (A).

In order to determine the local concentration of GM130 within the two forms of condensed GM130 phases, we measured the mean fluorescence intensity of recombinant GFP under the same experimental conditions and obtained a calibration curve (Fig. 6A). This calibration curve was linear over the whole range of intensities measured for GM130. Furthermore, because evaporation at the rim concentrates salt ions along with protein, we wanted to rule out that the observed phase separation was simply a result of the protein salting out by showing that the measured concentrations did not depend on the initial salt concentration in the buffer. We therefore varied the starting KCl concentration in the droplet from 0 to 500 mm and quantified the concentration of GM130 in the resultant condensates (Fig. 6B).

The concentration of GM130 in the droplets (region 1) was 150 ± 20 µm independent of [KCl] above ~ 100 mm and somewhat higher (170 ± 20 µm) at 0–100 mm KCl. In the continuous dense phase (region 2), the concentration of GM130 was 230 ± 30 µm independent of [KCl] above ~ 100 mm and marginally lower (200 ± 30 µm) at lower [KCl]. Although the GM130 concentration in the condensed droplet phases was largely independent of salt concentration and temperature, the size distribution of the droplets strongly depended on these variables (Fig. S4). These data therefore confirm that phase separation in the evaporation assay is not a result of salting out of the protein, but an intrinsic feature of the GM130.

### Possible implications for organization of the Golgi

Our results indicate that the Golgin GM130 indeed has the intrinsic capacity to phase separate into liquid‐like condensates. Liquid–liquid phase separations of polymers, including proteins, rely either on stochastic combinations of a large number of very low‐affinity intermolecular interactions among small clusters of side chains in the case of intrinsically disordered regions (IDR) of extensive low complexity sequence‐containing proteins 11, 29, 34, 35; or on multivalent moderate affinity interactions among folded scaffolding domains (such as with SH3 and SH2 domain‐containing proteins 36, 37). Because GM130 contains neither such sequences nor such domains, the molecular mechanism that allows for weak collective interactions among GM130 tetramers is unclear. Since most of the surface of GM130 (Fig. 1A) consists of helical bundles, it seems most likely that weak interactions involving these surfaces are critical determinants. The fact that phase separation occurs in the 50–100 µm protein concentration range at physiological ionic strength, pH, and temperature suggests that the underlying interactions are in this same range of affinities, and therefore likely involve individual side chains or small clusters of them located on the surface of the rods. This would be broadly analogous to condensates forming from IDRs and the key differences being that for GM130, the involved residues would be on the surface of a highly structured and rigid rod and that different side chains may be involved than those driving IDR condensation.

These *in vitro* observations seem likely to be relevant to the physical state of GM130 in the native Golgi stack for several reasons. First, analogous condensates can form in cells. Overexpressed GM130 is transported into the nucleus to form spherical condensates that are initially dynamic and liquid‐like. Second, these droplets evidently provide an environment, which is energetically similar to that experienced by endogenous GM130 as it resides in the Golgi, because endogenous GM130 efficiently relocates to join in them. Third, a straightforward estimate (Fig. S5) of the overall concentration of GM130 in the *cis* Golgi suggests that it is similar to its concentration within the condensates. Yet, there must also be significant differences because the endogenous GM130, though packed at similar overall density as it is in spherical condensates, is at least in part organized on the Golgi surface as an oriented monolayer by binding from one end to its protein receptor, GRASP65. It seems likely that even within the spherical condensates such lateral registration occurs locally and intrinsic to the mechanism of coacervation.

In conclusion, at least one member of the Golgin family undergoes liquid–liquid phase separation under conditions that approximate those in the cell. We speculate that most if not all Golgins and similarly structured vesicle tethers may also have this capacity. While there are many sequence similarities among the Golgins, they differ greatly in size. If lateral registration is an important principle underlying phase separation of this class of proteins, then length could be part of a code that enables the spontaneous assembly of domains within the Golgi having distinct Golgin compositions 38. Is the Golgi surface contained by a protective ‘cocoon’ of two‐dimensional Golgin condensates that excludes most cellular constituents (‘zone of exclusion’ 39, 40 but selectively admits vesicles containing the cognate Rab GTPase proteins, much as the nuclear pore admits its cargo into a hydrogel 34? It may even be that the Golgi—including its membranes—is templated by this cocoon rather than the other way around.

## Author contributions

AAR, PZ, AME, LCL, AHB, HZ, IL‐M, and FP analyzed data and performed experiments and contributed portions of the manuscript. AAR, AME, and JER designed research and wrote the manuscript.

## Supporting information


**Fig. S1.** (A) Quantitative western blots of endogenous GM130 and GRASP65 in Expi293F lysates compared to standards of their respective purified recombinant counterparts at known concentrations. (B) Quantitation of the western blots yields ~ 270 000 molecules/cell for GM130 and ~ 19 000 molecules/cell for GRASP65. (C) Hela cells were electroporated with mEGFP‐GM130 or left untransfected. 24 h post‐transfection, cells were fixed and immunolabeled with anti‐GPP130 (Biolegend 923801) followed by anti‐rabbit AF647 secondary Ab (Thermo Fisher Scientific A‐21244). Scale bars: 5 µm.
**Fig. S2.** A number of proteins chosen as negative controls do not phase separate in the evaporation assay.
**Fig. S3.** Different domains of GM130 exhibit different capacities to undergo phase separation.
**Fig. S4.** mEGFP fluorescence micrographs of the droplet evaporation assay showing phase‐separated mEGFP‐GM130‐FLAG (5 mm HEPES/KOH pH 7.3) near the rim for various initial [KCl] in the range 0–500 mm, as indicated, and at either 23 °C (left panel) or 37 °C (right panel).
**Fig. S5.** Estimate of the local concentration of GM130 at the *cis*‐Golgi for comparison with data shown in Fig. 5.
**Appendix S1.** Concentration Measurements of mEGFP Near the Rim.

**Video S1.** Origin of the coffee ring effect in an evaporating drop (blue) on a flat substrate (gray).

**Video S2.** Fluorescence confocal microscopy of recombinant mEGFP‐GM130‐FLAG in the droplet evaporation assay (20 mm HEPES/KOH pH 7.3, 140 mm KCl, 1 mm MgCl_2_, 1 mm DTT, and at 37° C).
