# Supplementary material for: Liquid–liquid phase separation of the Golgi matrix protein GM130
Source: FEBS Lett. 2019 Dec 26;594(7):1132–44. doi: 10.1002/1873-3468.13715 (PMC7160038; doi:10.1002/1873-3468.13715)
Supplement: Supplementary file 1 — Fig. S1. (A) Quantitative western blots of endogenous GM130 and GRASP65 in Expi293F lysates compared to standards of their respective purified recombinant counterparts at known concentrations. (B) Quantitation of the western blots yields ~ 270 000 molecules/cell for GM130 and ~ 19 000 molecules/cell for GRASP65. (C) Hela cells were electroporated with mEGFP‐GM130 or left untransfected. 24 h post‐transfection, cells were fixed and immunolabeled with anti‐GPP130 (Biolegend 923801) followed by anti‐rabbit AF647 secondary Ab (Thermo Fisher Scientific A‐21244). Scale bars: 5 µm. Fig. S2. A number of proteins chosen as negative controls do not phase separate in the evaporation assay. Fig. S3. Different domains of GM130 exhibit different capacities to undergo phase separation. Fig. S4. mEGFP fluorescence micrographs of the droplet evaporation assay showing phase‐separated mEGFP‐GM130‐FLAG (5 mm HEPES/KOH pH 7.3) near the rim for various initial [KCl] in the range 0–500 mm, as indicated, and at either 23 °C (left panel) or 37 °C (right panel). Fig. S5. Estimate of the local concentration of GM130 at the cis‐Golgi for comparison with data shown in Fig. 5. Appendix S1. Concentration Measurements of mEGFP Near the Rim. [file FEB2-594-1132-s001.pdf]

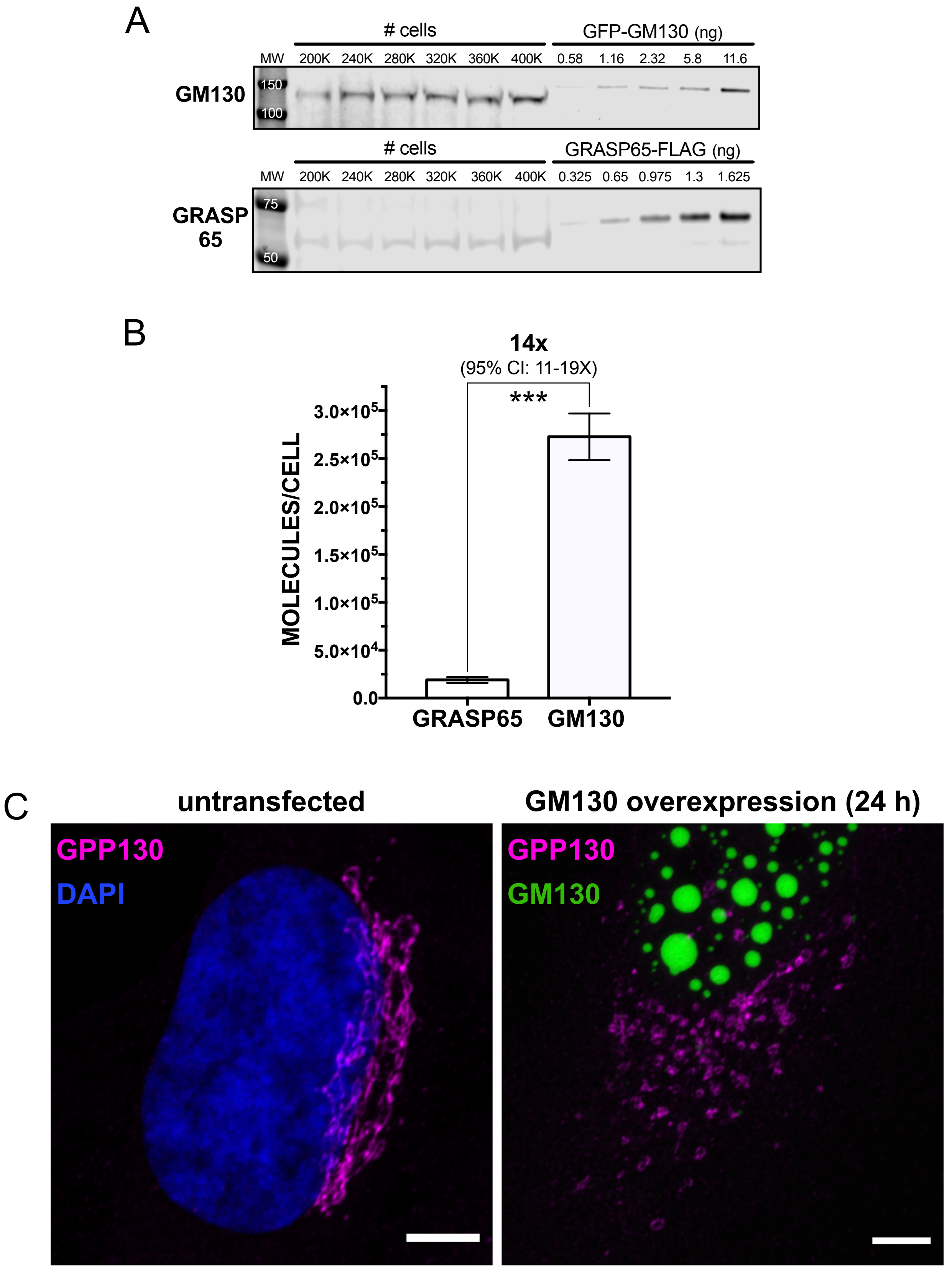

Supplemental Figure 1

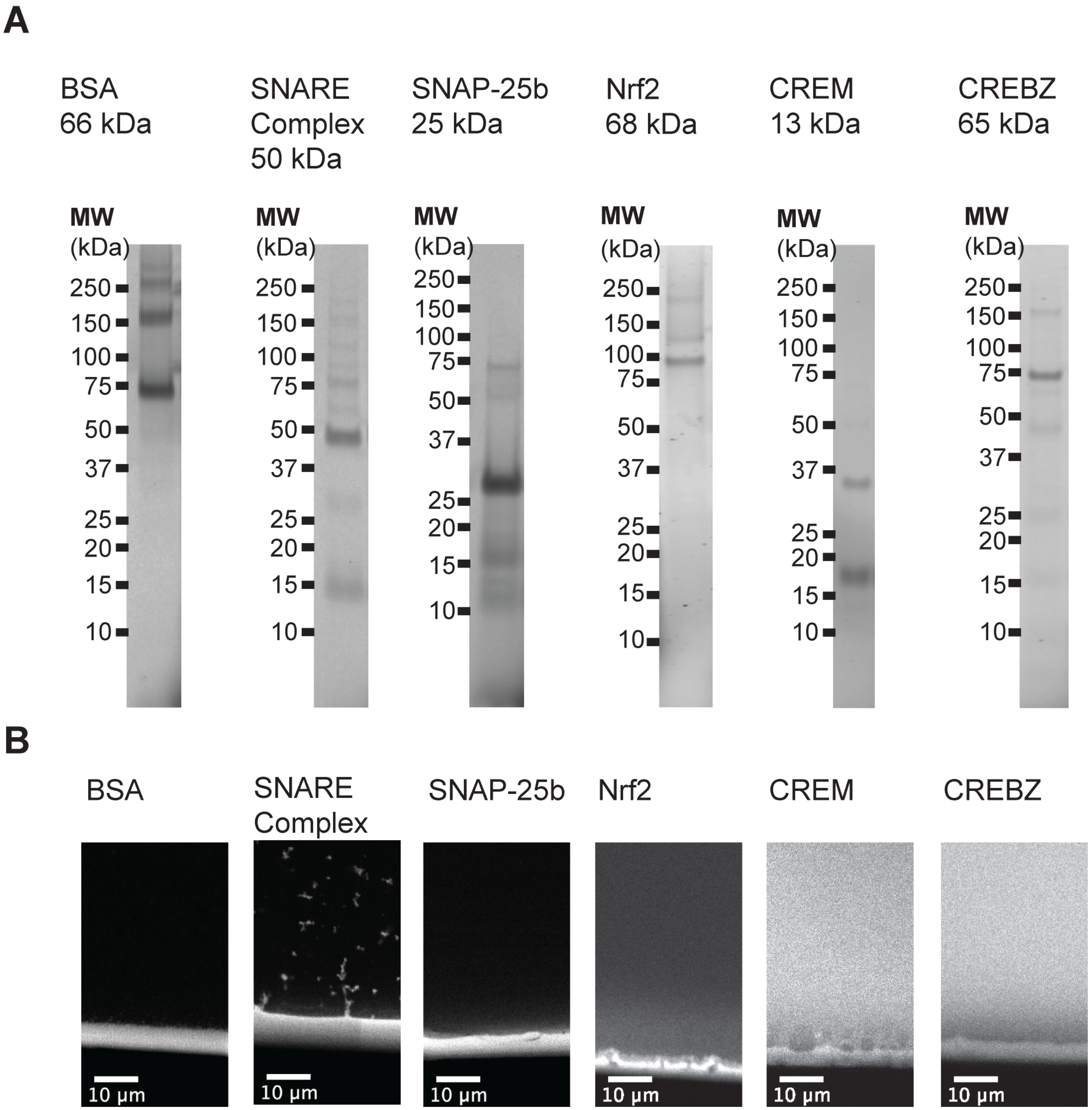

Supplemental Figure 2

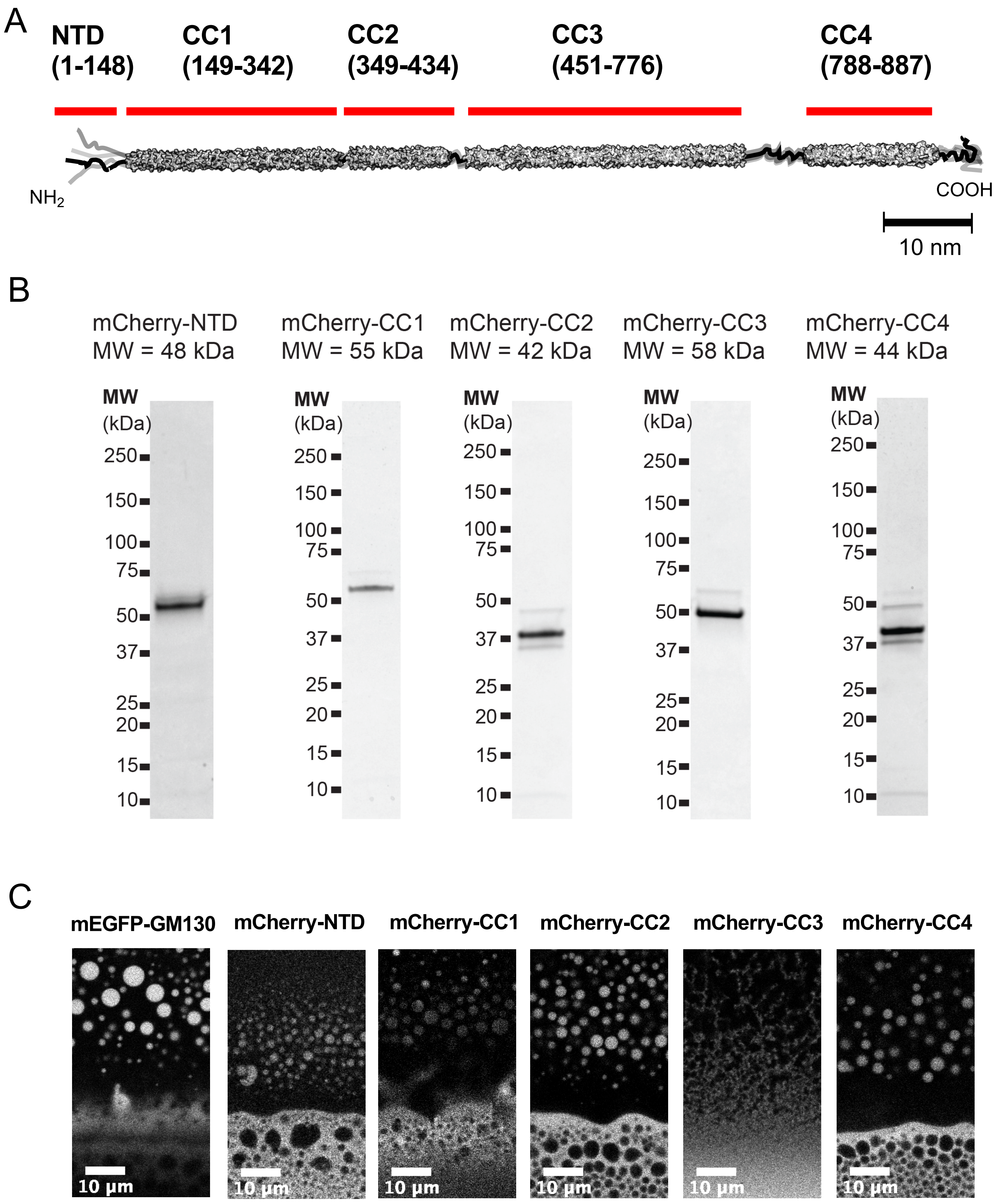

Supplemental Figure 3

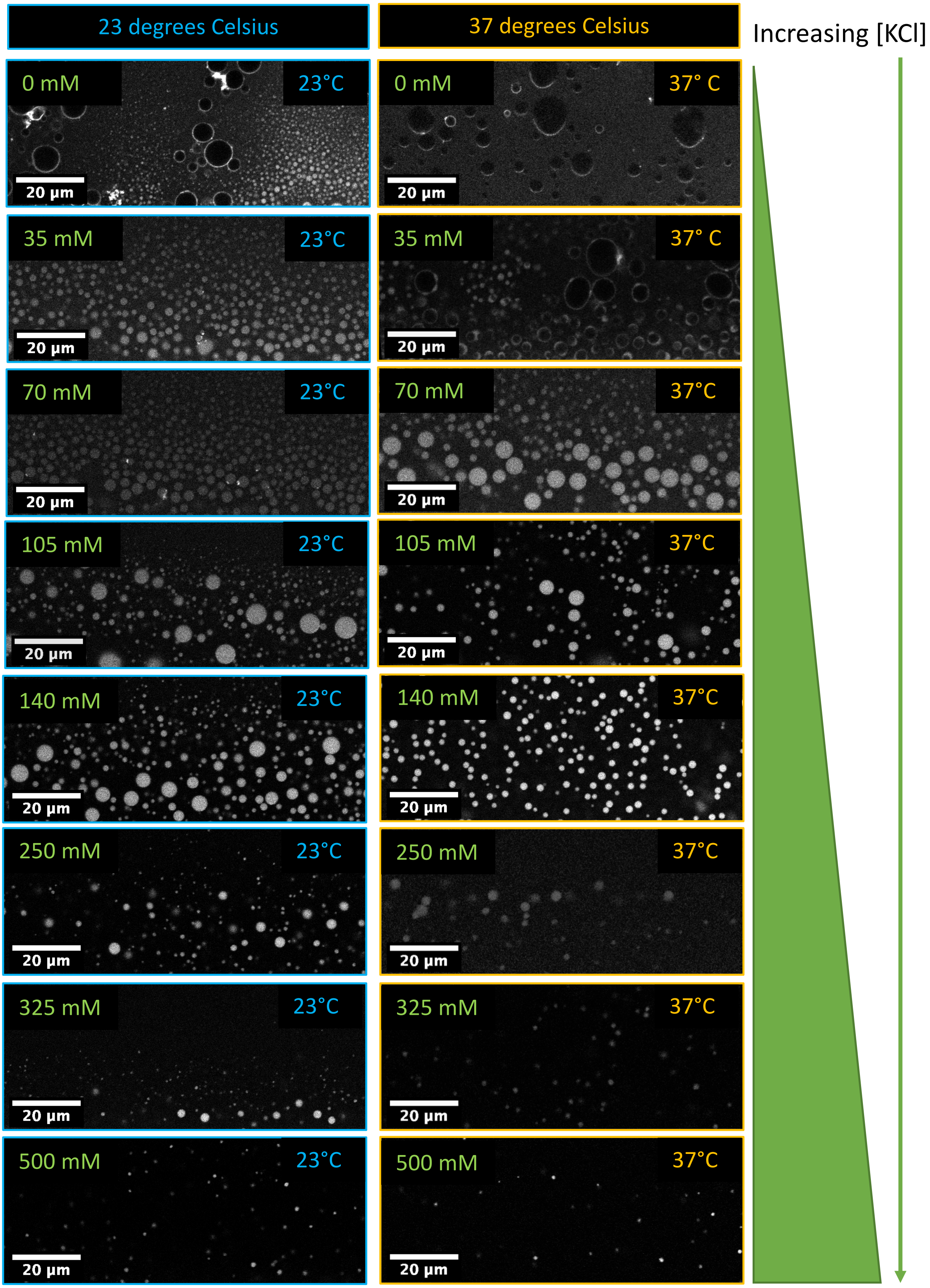

Supplemental Figure 4

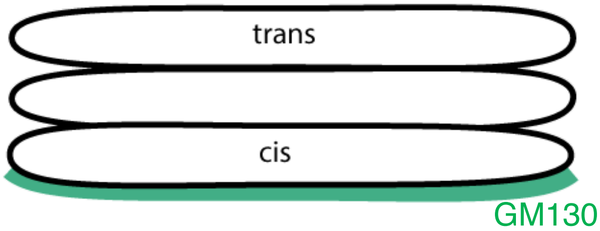

$S_{cis\text{-}Golgi} \approx 100\ \mu m^2$ ← ~100 ministacks/Golgi, ~1 μm diameter/ministack

$V_{GM130} \approx 50\ nm \times 100\ \mu m^2 = 5\ \mu m^3 = 5 \times 10^{-15}\ l$

↖ zone of exclusion

$n_{GM130/cell} \approx 0.5 \times 10^{-18}\ mol$ ← ~ 300,000 molecules/cell

$n_{GM130/cell}/V_{stack} \approx (0.5 \times 10^{-18}\ mol) / (5 \times 10^{-15}\ l) \approx \mathbf{100\ \mu M}$

↑

concentration of GM130
at the cis-Golgi

Supplemental Figure 5

APPENDIX S1

Concentration Measurements of mEGFP Near the Rim

In order to validate the coffee ring effect in our droplet evaporation assay, we measured the concentration of mEGFP near the rim of the drop during evaporation and compared it to a quantitative model. We deposited a 5 µL drop of 2.5 µM mEGFP solution (140 mM KCl, 5 mM HEPES/KOH pH 7.3, 1 mM $MgCl_2$, 1 mM DTT) on a microscope slide and measured the increase in fluorescence intensity within 120 µm of the drop rim over the course of evaporation using a confocal microscope, with the temperature held at 37 °C (**Fig. A1**). A continuous increase in fluorescence intensity was observed, as expected, given that fluorescence intensity is proportional to the protein concentration. We found that the fluorescence intensity near the rim increased in an

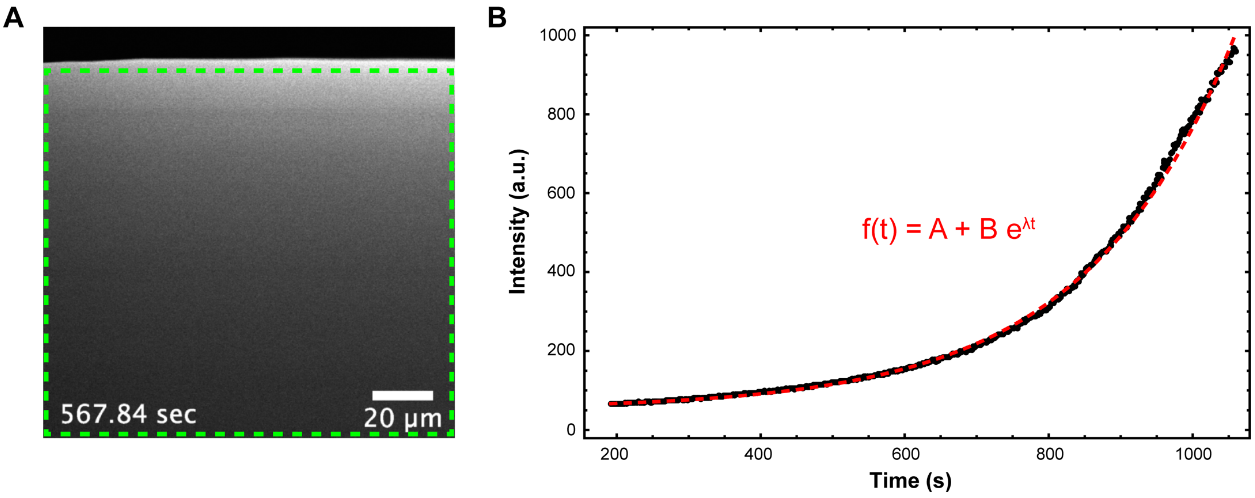

exponential manner, as evidenced by the exponential fit (**Fig. A1 B,** red).

Figure A1 Measurements of mEGFP fluorescence near the drop rim over the course of evaporation. A) Confocal image of mEGFP fluorescence near the rim of the drop at t = 567.84 s. Capillary flows induced by the coffee ring effect concentrate the protein near the rim. The green dashed line circumscribes the region of interest (ROI) within 120 µm of the drop rim, for which the average fluorescence intensity was measured and plotted in panel B. B) Time-dependence of the average fluorescence intensity within the green ROI in panel A. The red dashed lines shows the best-fit of the data with a single exponential function. The measurements start at 200 seconds due to time lag in depositing the droplet on the microscope slide and finding the focal plane on the microscope. After 1050 seconds, the droplet entered the advanced phase of drying out, and the fluorescence intensity started to decrease, likely due to denaturation of the mEGFP.

Theory of the Coffee Ring Effect

We compared this result with a quantitative model that takes as inputs the initial droplet volume V_0, initial protein concentration c_0, the time for the droplet to evaporate t_f, the diffusivity of water vapor in air at 37 °C, D_0, and the saturation and ambient concentrations of water vapor in air at 37 °C, Φ_{sat} and Φ_0, respectively. With these inputs, the evaporation, droplet geometry, and the evaporation-induced capillary flows were calculated based on the mathematical framework established by Deegan et al. [1]. Specifically, the droplet geometry is described as a spherical cap, with the drop height at time *t* and distance *r* from the center of the drop given by

$$h(r,t) = \sqrt{\frac{R^2}{\sin^2\theta} - r^2} - R\cot\theta, \quad (1)$$

where *R* is the radius of the contact line and θ is the contact angle (**Fig. A2**) [2].

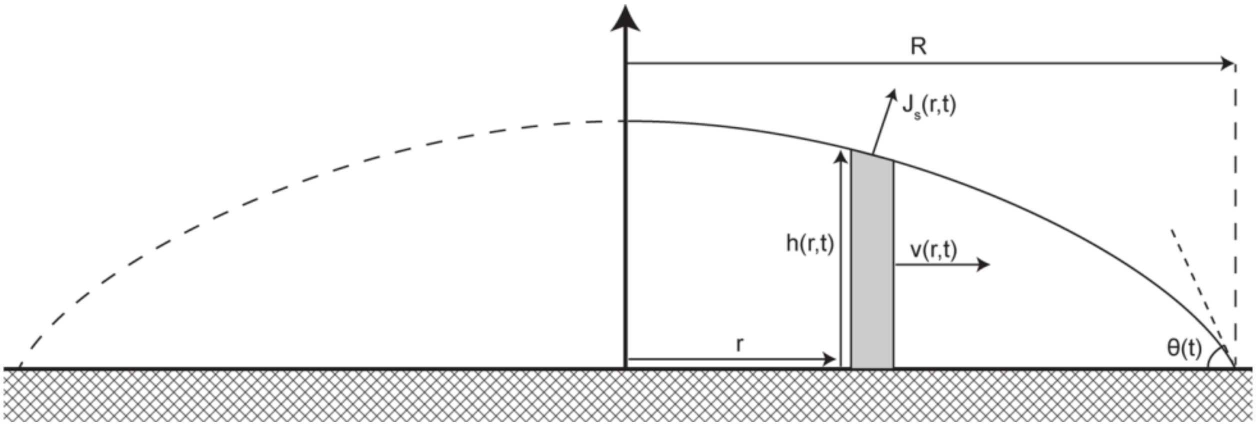

Figure A2 For all times t, the drop geometry is described by a spherical cap with contact angle $\theta(t)$ and contact line radius *R* (Eq. 1), where $h(r,t)$ is the height of the drop surface at distance *r* from drop center and at time t. $J_s(r,t)$ denotes the evaporative flux of solvent at the drop surface and $v(r,t)$ denotes the height-averaged velocity of solvent along *r*.

The contact line at $r = R$ is pinned, and only the droplet height, $h(r,t)$ for $r < R$, can change in response to evaporation of solvent (water). Consequently, the difference between the solvent volume lost due to evaporation during time *dt* in the annulus spanning *r* to $r + dr$, and the change in volume due to decrease in drop height in the same time interval must be compensated for by a radial flow $v(r,t)$. Specifically, given the positive evaporative flux of solvent from the drop surface, $J_s(r,t)$, the height-averaged radial velocity of solvent $v(r,t)$ is given by

$$v(r,t) = -\frac{1}{\rho r h}\int_0^r r'\left(J_s(r',t)\sqrt{1+\left(\frac{\partial h}{\partial r'}\right)^2} + \rho\frac{\partial h}{\partial t}\right)dr', \quad (2)$$

where ρ is the density of the solvent [3]. Once the velocity has been obtained, the trajectory of a solvent volume element starting at radius r_0 for $t = 0$ s is calculated by numerically integrating

$$\frac{dr(t)}{dt} = v(r,t), \quad (3)$$

with boundary condition $r(0) = r_0$. The time $t(r_0)$ required for the volume element to be transported to the rim ($r = R$) is given by numerically solving

$$r(t) = R. \quad (4)$$

In other words, by time $t(r_0)$, all solvent in the annulus spanning r_0 to R will have been transported to the rim (**Fig. A3**).

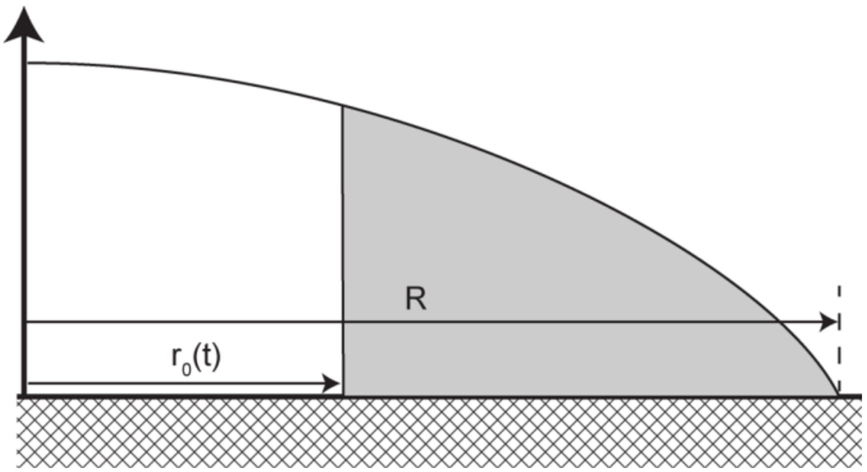

Figure A3 All volume elements with $r_0 \leq r < R$ at $t = 0$ s (gray) are transported to the rim in time $t(r_0)$.

To first approximation, molecular diffusion of the solute (protein) can be neglected because advection dominates the solute transport near the rim. Thus, we can directly calculate the time-dependence of solute concentration within the annulus $(R - \Delta r) \leq r \leq R$ using

$$c_{rim}\big(t(r_0)\big) = \frac{c_0 \int_{r_0}^{R} r' h(r',0)\, dr'}{\int_{R-\Delta r}^{R} r' h\big(r', t(r_0)\big)\, dr'}, \quad (5)$$

where c_0 is the solute concentration at time $t = 0$ s. Δr is chosen to match the ROI in **Fig. A1 A** (i.e. 120 μm).

Calculating the Evaporative Flux

In order to compute $v(r,t)$, we need to specify the evaporative flux $J_s(r,t)$. We calculated $J_s(r,t)$ from $\Phi(\mathbf{r})$, the spatial distribution of water vapor concentration surrounding the water drop using

$$J_s(r,t) = -D\nabla\Phi \cdot \mathbf{n}, \quad (6)$$

Where $\mathbf{n}$ is the normal vector to the drop surface. $\Phi(\mathbf{r})$ was determined by solving the Laplace's equation

$$\Delta\Phi = 0, \quad (7)$$

with Dirichlet boundary conditions of $\Phi = \Phi_{sat}$ at the drop surface and $\Phi = \Phi_0$ infinitely far away from the drop surface, and Neumann boundary conditions $\nabla \Phi \cdot \mathbf{n}_g = 0$ on the surface of the glass substrate, where $\mathbf{n}_g$ is the normal vector to the substrate. This problem is equivalent to finding the electrostatic potential outside of a lens-shaped charged conductor in free space, and can be solved analytically using toroidal coordinates α, β, φ, which are related to the cylindrical coordinates r, h, φ as follows [4]:

$$r = \frac{\lambda \sinh\alpha \cos\varphi}{\cosh\alpha - \cos\beta}, \quad (8a)$$

$$h = \frac{\lambda \sinh\alpha \sin\varphi}{\cosh\alpha - \cos\beta}, \quad (8b)$$

where

$$0 \leq \alpha < \infty, \qquad -\pi < \beta \leq \pi, \qquad -\pi < \varphi \leq \pi,$$

$\lambda > 0$ is a scale factor, and φ maps onto itself. Laplace's equation in toroidal coordinates has the form

$$\frac{\partial}{\partial\alpha}\left(\frac{\sinh\alpha}{\cosh\alpha-\cos\beta}\frac{\partial\Phi}{\partial\alpha}\right)+\frac{\partial}{\partial\beta}\left(\frac{\sinh\alpha}{\cosh\alpha-\cos\beta}\frac{\partial\Phi}{\partial\beta}\right)+\frac{1}{(\cosh\alpha-\cos\beta)\sinh\alpha}\frac{\partial^2\Phi}{\partial\varphi^2}=0. \tag{9}$$

By making the substitution

$$\Phi = \sqrt{2\cosh\alpha - 2\cos\beta}\,\Psi, \tag{10}$$

Eq. (9) becomes

$$\frac{\partial^2\Psi}{\partial\alpha^2}+\frac{\partial^2\Psi}{\partial\beta^2}+\coth\alpha\frac{\partial\Psi}{\partial\alpha}+\frac{1}{4}\Psi+\frac{1}{\sinh^2\alpha}\frac{\partial^2\Psi}{\partial\varphi^2}=0, \tag{11}$$

which is separable by setting

$$\Psi = A(\alpha)B(\beta)C(\varphi). \tag{12}$$

However, since the problem at hand has rotational symmetry, we can set $C(\varphi) = 1$ such that substituting Eq. (12) into Eq. (11) yields

$$\frac{1}{A}\frac{d^2A}{d\alpha^2}+\frac{\coth\alpha}{A}\frac{dA}{d\alpha}+\frac{1}{4}=-\frac{1}{B}\frac{d^2B}{d\beta^2}=n^2, \tag{13}$$

where n^2 is a constant, resulting in the equations

$$\frac{d^2B}{d\beta^2}+n^2B=0, \tag{14a}$$

$$\frac{1}{\sinh\alpha}\frac{d}{d\alpha}\left(\sinh\alpha\frac{dA}{d\alpha}\right)-\left(n^2-\frac{1}{4}\right)A=0. \qquad (14b)$$

Finally, using the substitution x = cosh(α), Eq. (14b) turns into Legendre's differential equation.

$$\frac{\mathrm{d}}{\mathrm{dx}}\left[(1-\mathrm{x}^2)\frac{\mathrm{dA}}{\mathrm{dx}}\right]+\left(\mathrm{n}^2-\frac{1}{4}\right)\mathrm{A}=0. \qquad (15)$$

Thus, the solution can be readily found and presented as a superposition of the solutions

$$\Phi_n=\sqrt{2\cosh\alpha-2\cos\beta}\left[a_nP_{n-\frac{1}{2}}(\cosh\alpha)+b_nQ_{n-\frac{1}{2}}(\cosh\alpha)\right]\times[c_n\cos n\beta+d_n\sin n\beta], \qquad (16)$$

where P_n and Q_n are the n-th order Legendre functions of the first and second kind, respectively, and a_n, b_n, c_n, and d_n coefficient determined by the boundary conditions. The solution for the equivalent problem of the electrostatic potential around a charged conductor with a shape defined by two intersecting spheres was derived by Lebedev [4], which translates to our problem as [2, 3]

$$\Phi(\alpha,\beta)=\Phi_0+(\Phi_{sat}-\Phi_0)\sqrt{2\cosh\alpha-2\cos\beta}\times\int_0^\infty P_{-\frac{1}{2}+I\tau}(\cosh\alpha)\frac{\cosh\theta\tau\cosh(2\pi-\beta)\tau}{\cosh\pi\tau\cosh(\pi-\theta)\tau}\,d\tau. \qquad (17)$$

Applying Eq. (6) yields the evaporative flux given by

$$J_s(r,t)=D_0\left(\frac{\sin\theta}{2}+(\cosh\alpha+\cos\theta)^{\frac{3}{2}}\right)\frac{(\Phi_{sat}-\Phi_0)}{R}\int_0^\infty P_{-\frac{1}{2}+\mathrm{I}\tau}(\cosh\alpha)\,\tau\frac{\cosh\theta\tau\tanh(\pi-\theta)\tau}{\cosh\pi\tau}, \qquad (18)$$

where r and t are implicitly defined by the definition of the toroidal coordinates and the time dependence of the contact angle, $\theta(t)$, respectively.

Calculation Results and Comparison with Experiment

Results of Eq. (18) for three different contact angles are shown in **Fig. A4**, using the values Φ_{sat} = 2.5 mM, Φ_0 = 1.65 mM, $D_0 = 5\cdot10^7$ μm^2 s^{-1}, and initial drop volume $V_0 = 5\cdot10^9$ μm^3 [5]. Note that

the drop geometry, and in particular the contact line radius R, are fully specified for contact angle θ and drop volume V_0 using Eq. (1). We find that the flux J_s diverges as r approaches R, and that this divergence becomes greater as θ decreases. This divergence is expected, since the vicinity of the rim is partially occupied by the substrate (glass surface), where $\Phi = 0$, thereby resulting in greater vapor concentration gradients. This contribution extends further into the drop center as the drop becomes flatter, thereby increasing the evaporative flux further from the rim as θ decreases.

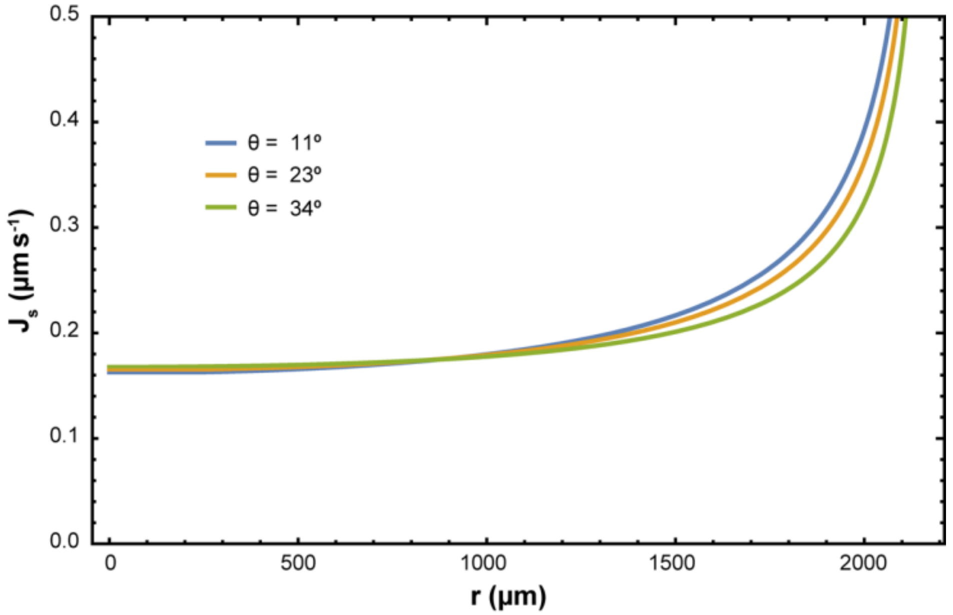

Figure A4 Evaporative flux plotted as a function of distance from the center of the drop for three contact angles θ. The flux in units µm s^{-1}, i.e. µm^3 water evaporated per µm^2 drop surface area per second.

This divergence has a direct effect on the radial velocity distribution $v(r,t)$, which also diverges near the rim. Thus, the concentration increase near the rim becomes faster with time as the drop dries out and θ tends to zero. To see this, we used the previously determined $J_s(r,t)$ to calculate the time-dependence of the drop shape, $h(r,t)$, over the course of evaporation by numerically solving

$$\rho \frac{d}{dt} \int_0^R r' h(r',t)\, dr' = \int_0^R r' J_s(r',t) \sqrt{1 + \left(\frac{\partial}{\partial r'} h(r',t)\right)^2}\, dr', \qquad (19)$$

where $\rho = 1$ mg ml^{-1} is the density of water and $h(r,t)$ and $J_s(r,t)$ depend implicitly on time via $\theta(t)$. We solved Eq. (19) for a number of initial contact angles ranging from 10° to 90° and found that

for θ(0) = 34°, the drop completely evaporated within t_f = 1135 s (i.e. $\theta(t_f) = 0°$) (**Fig. A5**), in good agreement with our experimental measurements (**Fig. A1 B**). We therefore used the initial condition θ(0) = 34° for all subsequent calculations. The time-dependence of $h(r,t)$ was calculated by substituting this solution into Eq. (1) and is shown in **Fig. A6**.

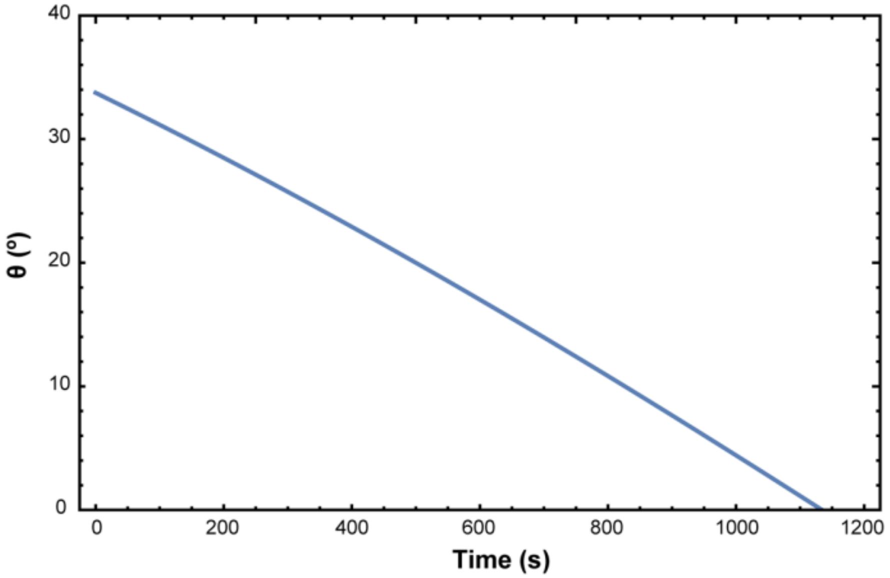

Figure A6 Time-dependence of the contact angle over the course of evaporation numerically calculated from Eq. (19) using the initial condition θ(0) = 34°. The time required for the drop to evaporate, 1135 s, agrees well with experimental measurements.

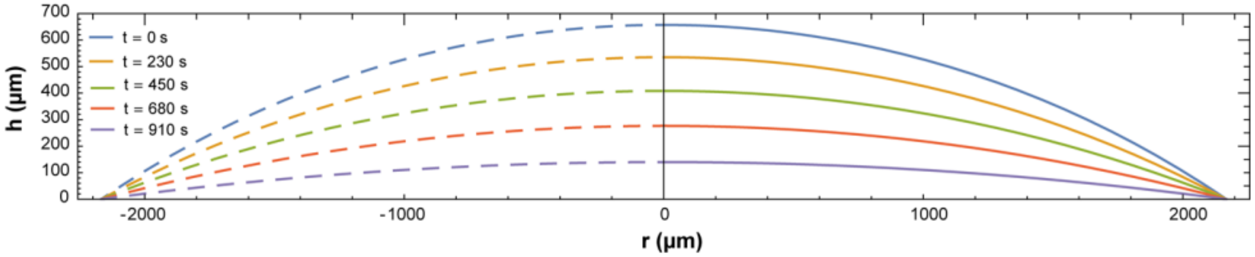

Figure A5 Drop geometry $h(r,t)$ plotted for five different time points by substituting the solution from Eq. (19) using the initial condition θ(0) = 34° into Eq. (1).

Having determined $h(r,t)$ and $J_s(r,t)$, we substituted these solutions into Eq. (2) to calculate $v(r,t)$ (**Fig. A7**). We then determined $t(r_0)$ using Eqs. (3) and (4), and calculated $c_{rim}(t)$ using Eq. (5) with $c_0 = 6$ and $\Delta r = 120$ μm (**Fig. A8**, green), which agrees well with experimental measurements for $t < 900$ s. The initial condition was chosen to reflect the measured data, extrapolated to $t = 0$ s. Deviations between the model and experiment for late time points ($t > 900$ s) stems like from

reduced mEGFP fluorescence under the unfavorable conditions present near the rim shortly before the drop has completely dried out. Thus, these model calculations recapitulate from first principles the expected concentration increase of protein near the drop rim as expected for the coffee ring effect, based on the experimental parameters of initial droplet volume V_0, initial protein concentration c_0, the time for the droplet to evaporate t_f, the diffusivity of water vapor in air at 37 °C, D_0, and the saturation and ambient concentrations of water vapor in air at 37 °C, Φ_{sat} and Φ_0, respectively.

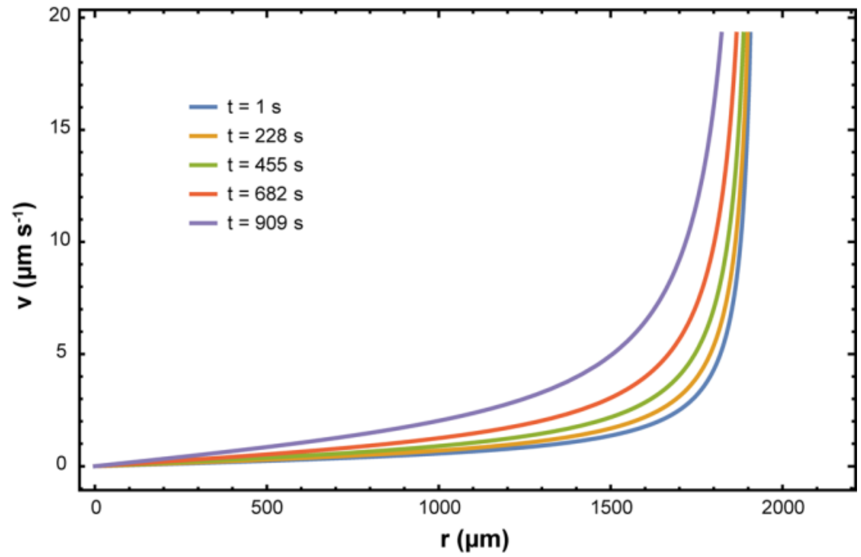

Figure A7 Height averaged radial velocity as a function of distance from the drop center, as numerically determined from Eq. (19), plotted for five different time points.

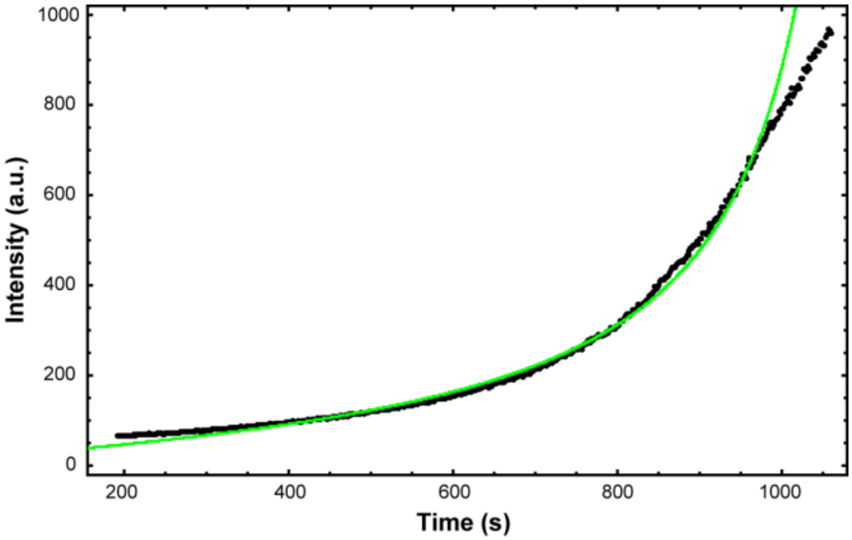

Figure A8 Comparison of experimental measurements (black) and model calculations (green) for the concentration increase within 120 μm of the rim (green ROI in **Fig. A1 A**). The ordinate has intensity units that reflect the measured values of mEGFP fluorescence, and are therefore proportional to protein concentration.

REFERENCES

1. Deegan, R. D., Bakajin, O., Dupont, T. F., Huber, G., Nagel, S. R. & Witten, T. A. (1997) Capillary flow as the cause of ring stains from dried liquid drops, *Nature.* 389, 827-829.

2. Hu, H. & Larson, R. G. (2002) Evaporation of a sessile droplet on a substrate, *J Phys Chem B.* 106, 1334-1344.

3. Deegan, R. D., Bakajin, O., Dupont, T. F., Huber, G., Nagel, S. R. & Witten, T. A. (2000) Contact line deposits in an evaporating drop, *Phys Rev E.* 62, 756-765.

4. Lebedev, N. N. & Silverman, R. A. (1965) *Special functions and their applications*, Rev. English edn, Prentice-Hall, Englewood Cliffs, N.J.,.

5. (1977) CRC handbook of chemistry and physics in pp. volumes, CRC Press, Cleveland, Ohio.
