# Supplementary material for: Liquid–liquid phase separation of the Golgi matrix protein GM130
Source: FEBS Lett. 2019 Dec 26;594(7):1132–44. doi: 10.1002/1873-3468.13715 (PMC7160038; doi:10.1002/1873-3468.13715)
Supplement: Supplementary file 4 [file FEB2-594-1132-s004.docx]

Supplemental Figure 1. (A) Quantitative western blots of endogenous GM130 and GRASP65 in Expi293F lysates compared to standards of their respective purified recombinant counterparts at known concentrations. (B) Quantitation of the western blots yields ~270,000 molecules/cell for GM130 and ~19,000 molecules/cell for GRASP65. (C) Hela cells were electroporated with mEGFP-GM130 or left untransfected. 24 hours post-transfection, cells were fixed and immunolabeled with anti-GPP130 (Biolegend 923801) followed by anti-rabbit AF647 secondary Ab (Thermo Fisher Scientific A-21244). Scale bars: 5 µm.

Supplemental Figure 2. A number of proteins chosen as negative controls do not phase separate in the evaporation assay. (A) In-gel fluorescence images showing SDS/PAGE of Alexa Fluor 647-labelled control proteins. BSA was chosen as an exemplary globular protein. The cytosolic domain of the SNARE complex was chosen because of its tetrameric coiled-coil structure that resembles the tetrameric coiled-coil domains of GM130. SNAP-25b was chosen as a monomeric coiled-coil constituent containing heptad repeats, a motif abundant in GM130. The transcription factors Nrf2, CREM, and CREBZ were chosen as exemplary coiled-coils, structurally similar to those found in GM130. (B) Confocal fluorescence micrographs show that none of the Alexa Fluor 647-labelled control proteins phase-separated upon evaporation in 5 mM HEPES/KOH pH 7.3, 140 mM KCl, 1 mM DTT, and 1 mM Mg2+, at 37°C. Scale bars: 10 µm.

Supplemental Figure 3. Different domains of GM130 exhibit different capacities to undergo phase separation. (A) Domain map of GM130. Based on the Multicoil2 coiled-coil prediction algorithm [38], GM130 was subdivided into five domains: one disordered N-terminal domain (NTD), and four coiled-coil domains (CC1-CC4). Residue numbers are shown below the respective domain names. (B) Coomassie-stained SDS/PAGE of the five GM130 domains defined in (A), purified as recombinant constructs with an N-terminal mCherry tag and a C-terminal FLAG tag. Molecular weights (MW) calculated based on amino acid composition are shown below the respective construct. (C) Confocal mCherry fluorescence micrographs of the recombinant GM130 domains upon evaporation in 5 mM HEPES/KOH pH 7.3, 140 mM KCl, 1 mM DTT, and 1 mM Mg2+, at 37°C. Phase separation into spherical condensates was observed for the NTD, CC2, CC4, and to a lesser degree for CC1, but not for CC3. As a reference, a fluorescence image for phase-separated full-length mEGFP-GM130-FLAG under the same conditions is shown on the left. Scale bars: 10 µm.

Supplemental Figure 4 mEGFP fluorescence micrographs of the droplet evaporation assay showing phase-separated mEGFP-GM130-FLAG (5 mM HEPES/KOH pH 7.3) near the rim for various initial [KCl] in the range 0 – 500 mM, as indicated, and at either 23 °C (left panel) or 37 °C (right panel). Scale bars: 20 µm.

Supplemental Figure 5. Estimate of the local concentration of GM130 at the cis-Golgi for comparison with data shown in Fig. 5. According to quantitative mass spectrometry data available on GM130 [16], there are roughly 300,00 copies of GM130 per cell, which we independently confirmed by quantitative western blotting (Fig. S1) Considering that GM130 is distributed over the entire cis-face [39] and that the zone of ribosome exclusion around the Golgi is 50 nm [40] yields, to a first approximation, a local concentration of 100 µM at the cis-Golgi, which agrees well with the phase-separated regime as determined for GM130 in vitro (Fig. 5).

Movie S1 Origin of the coffee ring effect in an evaporating drop (blue) on a flat substrate (gray). The contact line along the interface between the drop surface and the substrate remains fixed during evaporation (pinned edges). Volume elements within the drop corresponding to spheroidal shells are colored in different shades of blue to show how solvent is transported to the rim during evaporation.

Movie S2 Fluorescence confocal microscopy of recombinant mEGFP-GM130-FLAG in the droplet evaporation assay (20 mM HEPES/KOH pH 7.3, 140 mM KCl, 1 mM MgCl2, 1 mM DTT, and at 37 °C). mEGFP-GM130-FLAG crosses the liquid–liquid phase separation boundary at 914 seconds, forming spherical condensates (left) and a continuous dense phase (right) within 1 second of the phase transition. Scale bar: 3 µm.
